# Monte Carlo Simulations for Dosimetry in Prostate Radiotherapy with Different Intravesical Volumes and Planning Target Volume Margins

**DOI:** 10.1371/journal.pone.0159497

**Published:** 2016-07-21

**Authors:** Wei Lv, Dong Yu, Hengda He, Qian Liu

**Affiliations:** 1 Britton Chance Center for Biomedical Photonics, Wuhan National Laboratory for Optoelectronics, Huazhong University of Science and Technology, Wuhan, China; 2 MoE Key Laboratory for Biomedical Photonics, Department of Biomedical Engineering, Huazhong University of Science and Technology, Wuhan, China; ENEA, ITALY

## Abstract

In prostate radiotherapy, the influence of bladder volume variation on the dose absorbed by the target volume and organs at risk is significant and difficult to predict. In addition, the resolution of a typical medical image is insufficient for visualizing the bladder wall, which makes it more difficult to precisely evaluate the dose to the bladder wall. This simulation study aimed to quantitatively investigate the relationship between the dose received by organs at risk and the intravesical volume in prostate radiotherapy. The high-resolution Visible Chinese Human phantom and the finite element method were used to construct 10 pelvic models with specific intravesical volumes ranging from 100 ml to 700 ml to represent bladders of patients with different bladder filling capacities during radiotherapy. This series of models was utilized in six-field coplanar 3D conformal radiotherapy simulations with different planning target volume (PTV) margins. Each organ’s absorbed dose was calculated using the Monte Carlo method. The obtained bladder wall displacements during bladder filling were consistent with reported clinical measurements. The radiotherapy simulation revealed a linear relationship between the dose to non-targeted organs and the intravesical volume and indicated that a 10-mm PTV margin for a large bladder and a 5-mm PTV margin for a small bladder reduce the effective dose to the bladder wall to similar degrees. However, larger bladders were associated with evident protection of the intestines. Detailed dosimetry results can be used by radiation oncologists to create more accurate, individual water preload protocols according to the patient’s anatomy and bladder capacity.

## Introduction

Stability of the target volume and radiotoxicity to organs at risk (OAR) are two principal factors that should be considered in radiotherapy planning. In prostate radiation treatment, the bladder is located above the region to be irradiated, and its shape and volume change significantly. Moreover, an increase of the bladder volume alters its shape, which may cause pronounced and unpredictable prostate displacement [1–4]. Conversely, if the bladder volume decreases, the radiation dose absorbed by the bladder wall, small intestine, and rectum increases, which may lead to serious organ toxicity [1,5,6].

In order to maintain stable interfraction and intrafraction volumes of the bladder, patients are prepared by preloading a certain volume of water to either fill or void the bladder before prostate radiation treatment [7]. However, the ability to fill or void the bladder differs remarkably among patients, and therefore the dose constraint is difficult to meet [6,8]. Image-guided radiotherapy (IGRT) restricts OAR toxicity by tracking the position of tumors and further reducing planning target volume (PTV) margin [9]. Nevertheless, because of the radiation toxicity and difficulty in maintaining and measuring the intravesical volume, few clinical studies have performed quantitative dosimetry with different bladder volumes and PTV margins. Hence, anthropomorphous computational models have been developed to simulate the dosimetry in radiation treatment.

The Medical Internal Radiation Dose committee (MIRD) has recognized the limitation of the constant-volume bladder model in dosimetry evaluation of internal radiation and constructed a deformable mathematical sphere model to simulate dosimetry of radiation from radioactive particles [10]. The committee concluded that the spherical approximation is appropriate for the bladder because the radiation dose to the bladder wall from non-penetrating radiation (the major fraction of total internal radiation dose) is relatively independent of the actual bladder shape [11]. However, photons have energy high enough to penetrate the organs in external radiotherapy. Accordingly, the dose to OAR is clearly affected by bladder shape, and a simple mathematical sphere model is not suitable for calculating dose distribution in radiotherapy.

Finite element (FE) analysis is widely applied in biomechanics to predict organ deformation and displacement. In FE analysis, a continuous problem domain can be decomposed into simpler parts called FEs, and the calculus of variations can be applied to solve the problems by minimizing the associated error function. Chai *et al*. [12,13] employed the FE method to simulate bladder deformation, but their bladder wall model was constructed by uniformly shrinking the outer bladder outline by 3 mm because of insufficient MRI image resolution. Krywonos *et al*. [14] used high-resolution MRI images to construct an FE model of the bladder. These researchers indicated that FE analysis can be potentially used for predicting bladder deformation.

A Chinese adult male computational phantom named Visible Chinese Human (VCH) was previously developed on the basis of a high-quality cryo-sectional image set, with a resolution of 0.1 mm × 0.1 mm × 0.2 mm [15,16]. In this study, the VCH voxelized model was converted to an FE model to perform organ deformation and establish pelvic models with different bladder volumes. The FE model was then converted back to a voxelized model and utilized in 3D conformal radiation therapy (3D-CRT) simulation. The dose to the organs was calculated to assess the effect of bladder volume and PTV margin on dose distribution.

## Materials and Methods

**Fig 1:**
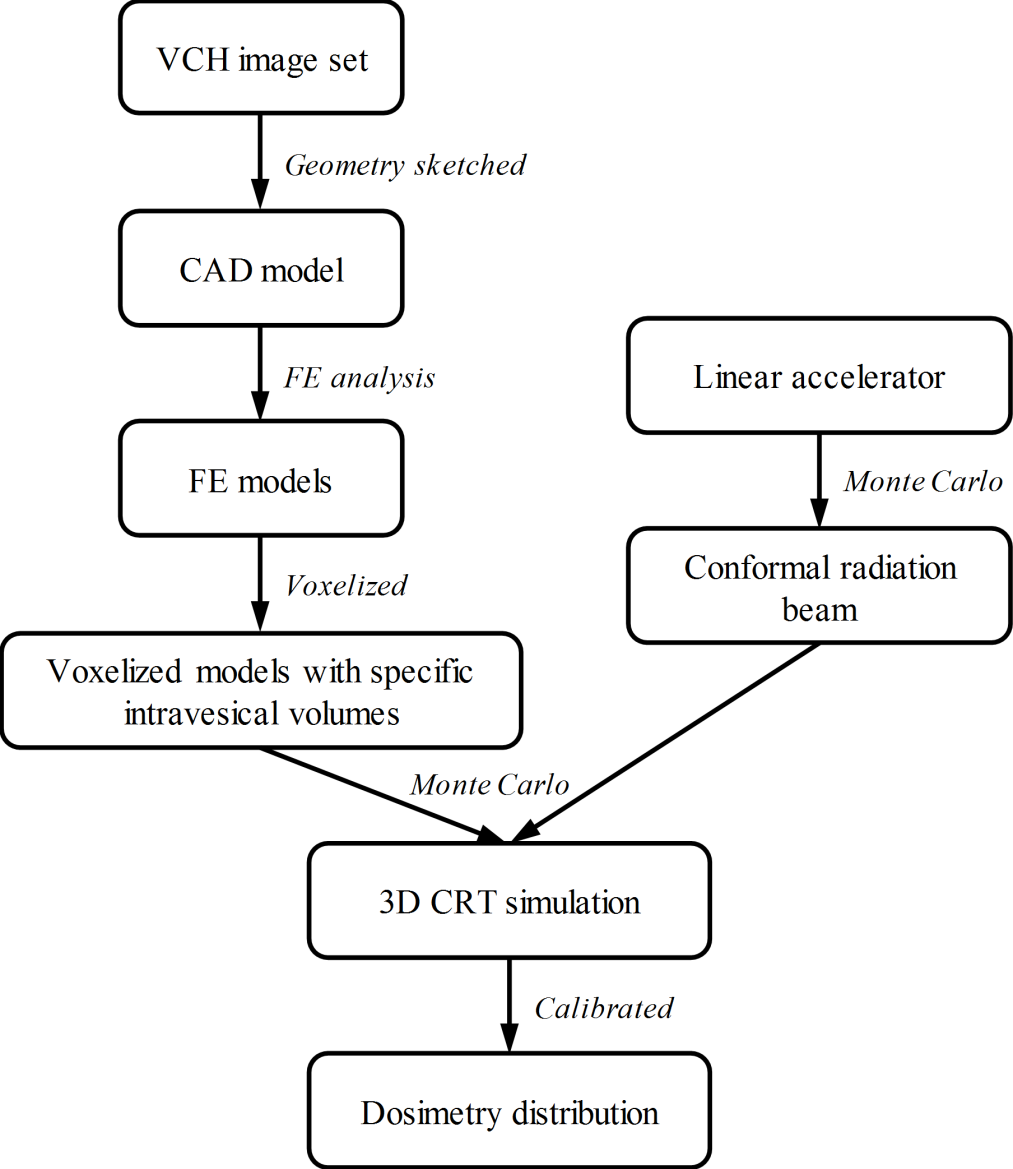
Flowchart of organ deformation and radiotherapy simulation.

The overall workflow of this study is presented in Fig 1, including the organ deformation and radiotherapy simulation approaches.

### Organ deformation

The tomographic image set used to develop the VCH computational phantom was obtained from high-quality cryo-sectional color photographs of an adult Chinese male cadaver (age, 24 years; height, 166 cm; weight, 58 kg). Image acquisition, segmentation, and 3D reconstruction were performed as previously described [15]. The resolution was reduced to 0.5 mm × 0.5 mm × 0.5 mm, and only the pelvic part was extracted from the whole VCH body to reduce computation time. The extracted pelvic model consisted of the bladder wall, prostate, bone, seminal vesicles, and part of the intestines. Considering that the small intestine and the rectum were both recognized as parts of the intestines in the previous VCH segmentation work, we combined doses to these organs in the present study.

A voxelized model cannot be directly applied in FE analysis; hence, the geometry and shape of soft tissues were sketched using a computer-aided design software (CATIA version 5, Dassault Systemes). The geometries of the organs were exported as solid models. The solid models were then subtracted from the VCH outline model to obtain the surrounding tissues, including muscle and fat, which are referred to as “body” in the following description.

**Fig 2:**
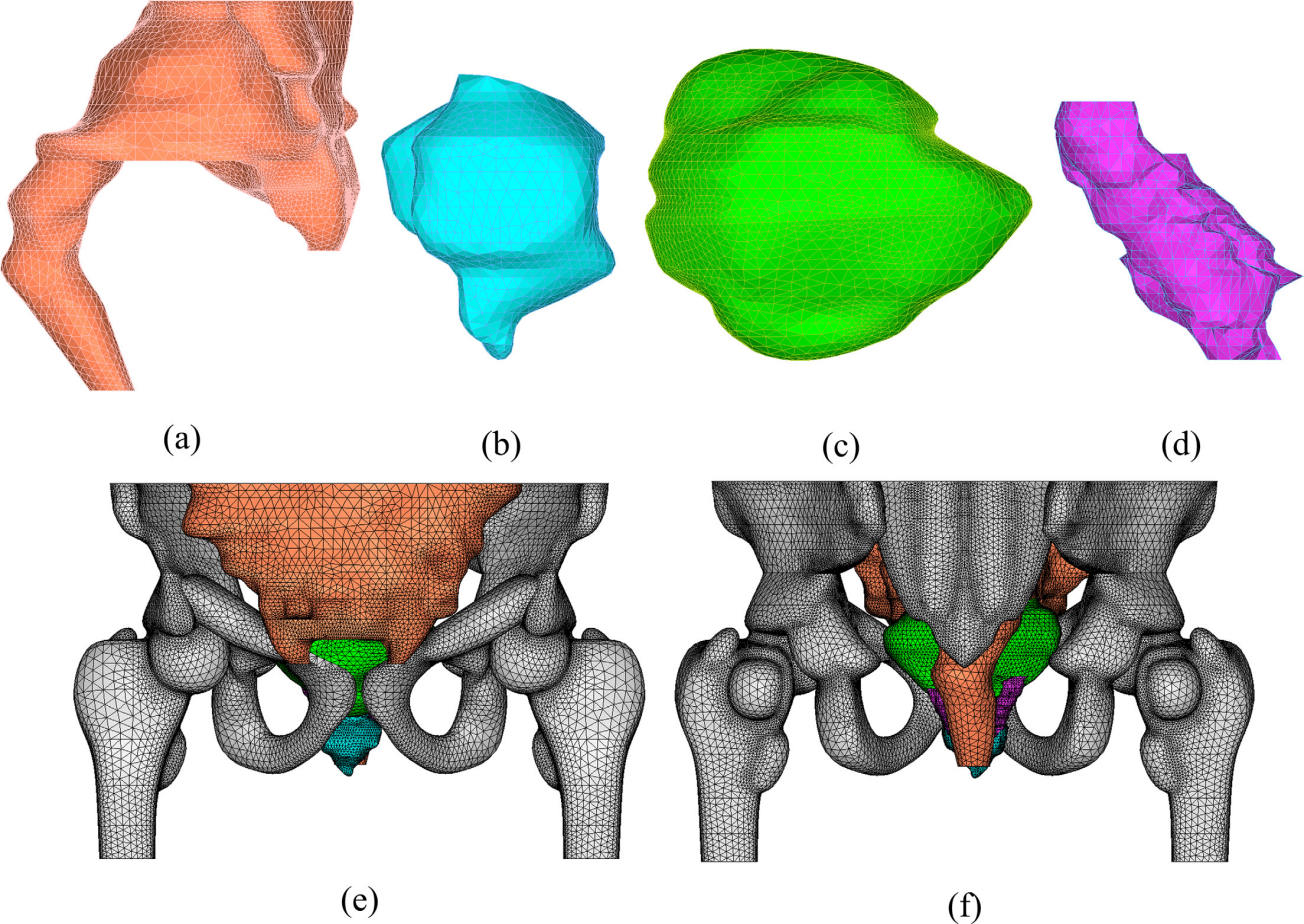
FE models constructed in Hypermesh. Right views of (a) intestines, (b) prostate, (c) bladder, and (d) seminal vesicles. (e) Anterior view and (f) posterior view of the VCH pelvic part.

The solid models of the pelvic organs were imported into an FE analysis software package (Hypermesh version 12, Altair) and meshed into tetrahedral elements. Fig 2 illustrates the solid models of each organ from the VCH and FE models after the meshing. Interfaces and material properties were subsequently assigned to each organ. Organ deformation and interaction were considered linear elastic processes, which are characterized by Young’s modulus *E* and Poisson’s ratio *v*. A larger Young’s modulus indicates a stiffer material, and a larger Poisson’s ratio corresponds to a less compressible tissue. The material properties were obtained from previous studies [12,17] and are shown in Table 1. The elements at the bottom of the whole model and the whole bone were constrained in the FE analysis because the inferior surface of the pelvic part and bone in the present study were assumed immobile during bladder filling.

**Table 1:** Properties of the organs in the pelvic region.

Organ	Young’s modulus (kPa)	Poisson ratio
Prostate	21	0.40
Bladder wall	10	0.50
Intestines	10	0.50
Seminal vesicles	11	0.40
Bone	1000	0.50
“Body”	15	0.40

A static kinematic analysis step was included in the FE analysis because bladder filling is a slow process. Chai *et al*. [12] assumed that bladder filling is the only cause of deformation of the pelvic region and applied isotropic pressure on the inner bladder wall; the results showed that this approach can be used to predict bladder deformation. Therefore, bladders with different intravesical volumes were constructed in this study by adjusting the uniform force magnitude. During bladder voiding, the direction of force was reversed to create a negative-pressure environment caused by urination.

After the FE simulation was completed, a series of pelvic tetrahedral models with different intravesical volumes was stored in a tetrahedral vertex and vertex coordinate format. An in-house program was developed to disassemble the FE model into tetrahedral elements and to extract each voxel inside the tetrahedron whose coordinates were divisible by 0.5. After the tetrahedrons were traversed and the extracted voxels were assigned to the referred organ index, the corresponding voxelized model was considered ready for Monte Carlo simulation.

### Radiotherapy simulation

This study mainly investigated the effects of organ deformation and displacement on dose distribution; therefore, a six-field coplanar 3D-CRT rather than intensity-modulated radiation therapy was used to avoid the variability of beam optimization. The whole prostate was considered as the clinical target volume (CTV). Two PTVs with 5- and 10-mm 3D margins were constructed around the CTV region.

The radiotherapy simulation was performed using the EGSnrc/BEAMnrc Monte Carlo code system [18,19]. The density and elemental composition of the organs were obtained from the International Commission on Radiological Protection (ICRP) publications 70 [20] and 89 [21]. A 6-MV XHA600 (Shinva Medical, Shandong, China) linear accelerator was modeled using the BEAMnrc software package to generate conformal radiation beams. Multileaf collimator position was adjusted according to the projection of the prostate from different angles. The energy, position, and direction of the output particles were recorded at the exit window of the accelerator as a phase space file. Dose distribution was determined using DOSXYZnrc [22] of the EGSnrc system with the accelerator phase space file and the voxelized phantom as the input.

The simulation in this study was performed using a workstation with an Intel Xeon 2.27 GHz CPU, 8 GB of RAM, and a 64-bit version of Windows Server 2008. A total of 5 × 10^8^ particles were tracked in each simulation to reduce the dose uncertainty in the region of interest to 10%.

Effective dose (*D*_eff_) and volume fraction that received more than 50 Gy (V50) were used in this study to describe the dose distribution. The dose and corresponding volume fraction were assumed to satisfy a power law relationship [23]; accordingly, *D*_eff_ was calculated using the following equation:

$$D_{eff}={\left(\sum D_{i}^{1/n}V_{i}\right)}^{n}$$

where *D*_*i*_ is the deposit dose in partial volume *V*_*i*_ and *n* is the tolerance parameter of the radiation dose of an organ; *n* of the bladder wall is 0.5 [24].

## Results

### Organ deformation

The mass of the bladder wall remained constant during the FE analysis; however, it changed in the course of reducing the resolution of the VCH and reverse modeling to the FE model. The masses of the original VCH bladder wall and the voxelized FE model were 51.11 g [16] and 52.23 g, respectively. The deviation between these values did not exceed 2.5%.

**Fig 3:**
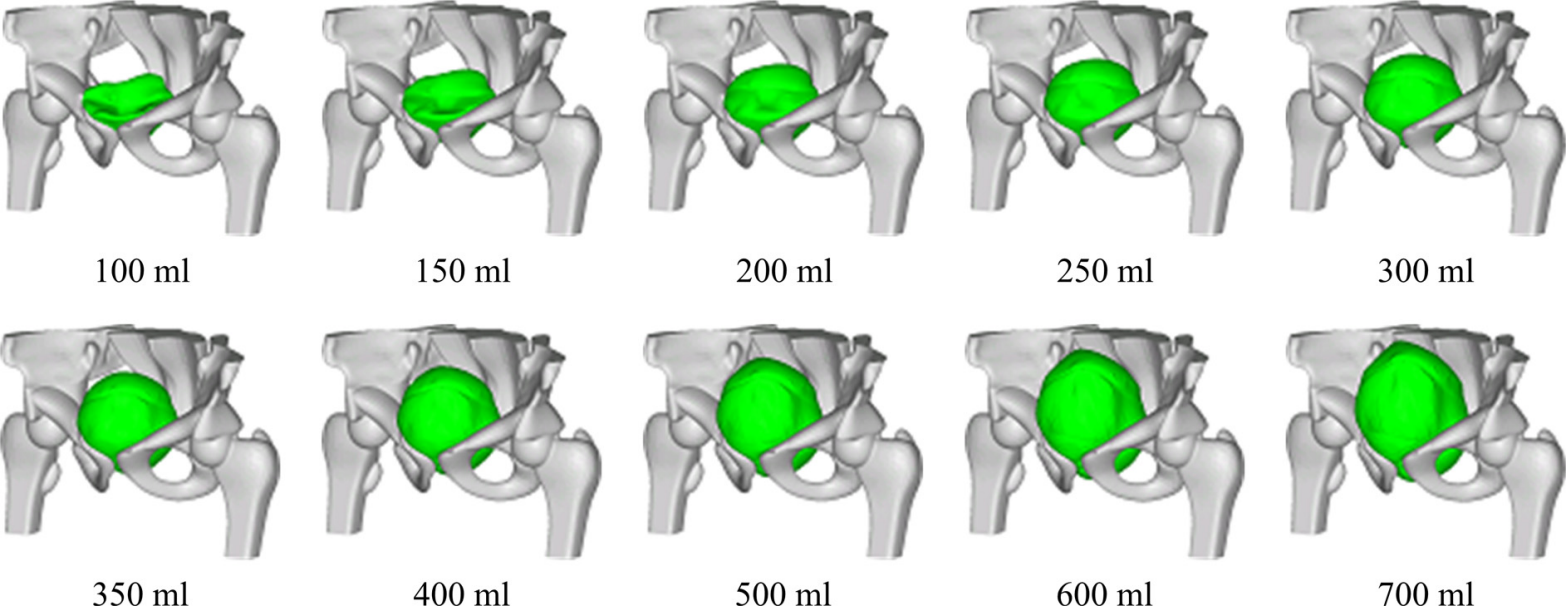
Ten pelvic models with different bladder volumes. Only the bladder and bone are shown for easier comparison.

**Fig 4:**
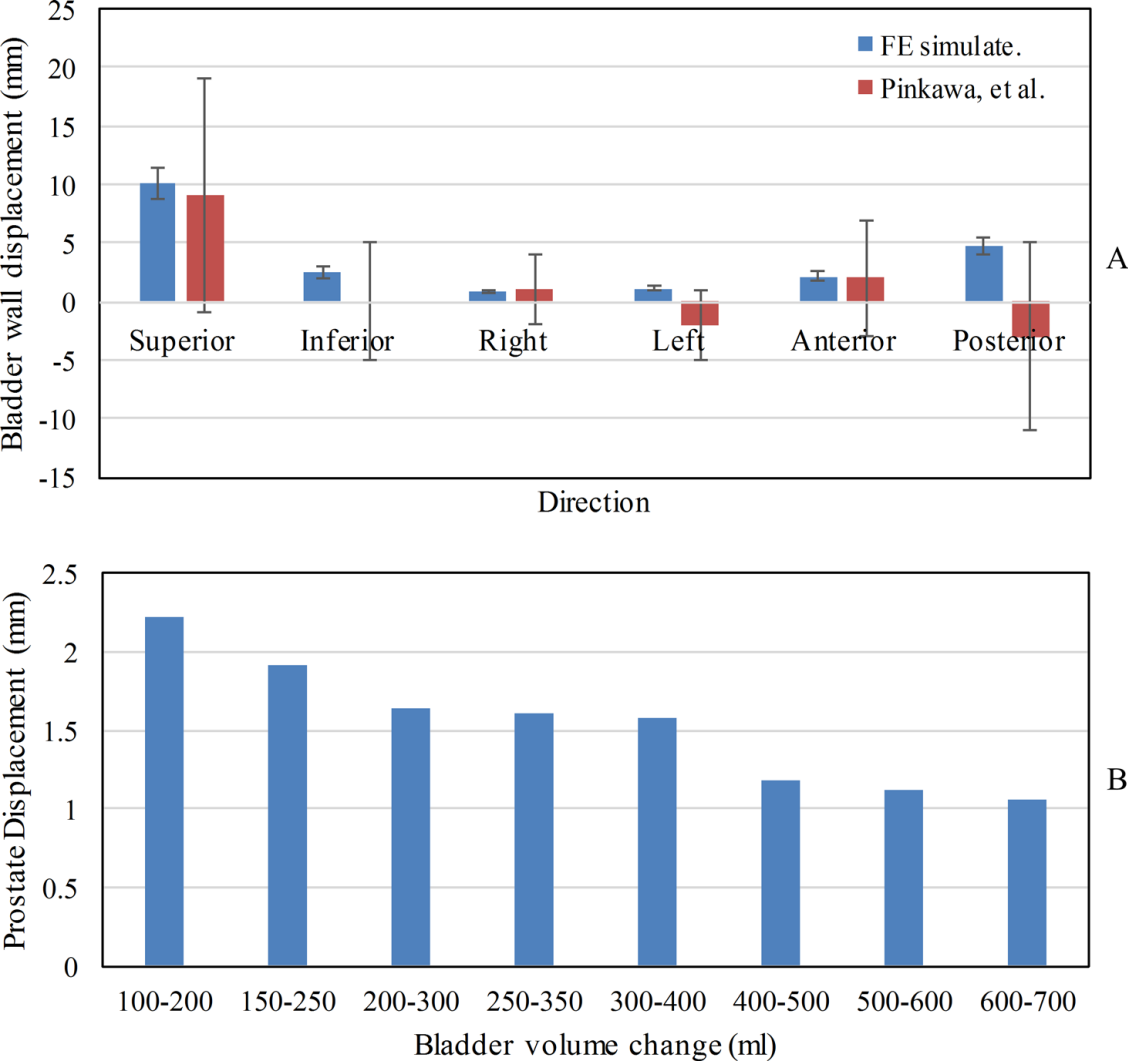
(A) Bladder wall displacement after a 100 ml increase in bladder volume. The average values and standard deviations of the simulated results and clinical results of Pinkawa *et al*. [25] are compared. (B) Prostate displacement after a 100-ml increase in bladder volume.

We constructed 10 pelvic models with intravesical volumes of 100, 150, 200, 250, 300, 350, 400, 500, 600, and 700 ml (Fig 3) to represent void bladders and bladders filled to different extents. The results of the FE analysis showed that the bladder wall mainly extended along the anterior–posterior and superior–inferior directions during bladder filling. The results for bladder wall displacement were compared with the clinical measurements reported by Pinkawa *et al*. [25], who performed computed tomography (CT) in 50 patients to analyze the displacements of the bladder wall during radiotherapy (Fig 4A). The comparison revealed that the two data sets were similar, which suggested that the pelvic models were suitable for the following radiotherapy simulations. The extended bladder wall pushed the small intestine upward and the rectum backward; the extended bladder wall also pushed the prostate from anterior–superior to posterior–inferior. The prostate shifted by 1–2.5 mm when the intravesical volume increased by 100 ml (Fig 4B).

### Dosimetry in prostate radiotherapy

The prescription dose in each radiotherapy simulation was 70 Gy to 95% of the PTV, and the absorbed dose to the voxels was scaled to satisfy the PTV coverage. As the radiation beam consistently targeted the prostate in each simulation, the mean dose to the prostate was 75 Gy, and the maximal dose did not exceed 125% of the prescription dose; these parameters were independent of the bladder volume and the PTV margin. The maximal dose to the bladder wall was approximately 80 Gy throughout the simulation; this finding indicated that bladder filling did not squeeze the bottom of the bladder wall out of the target volume.

**Fig 5:**
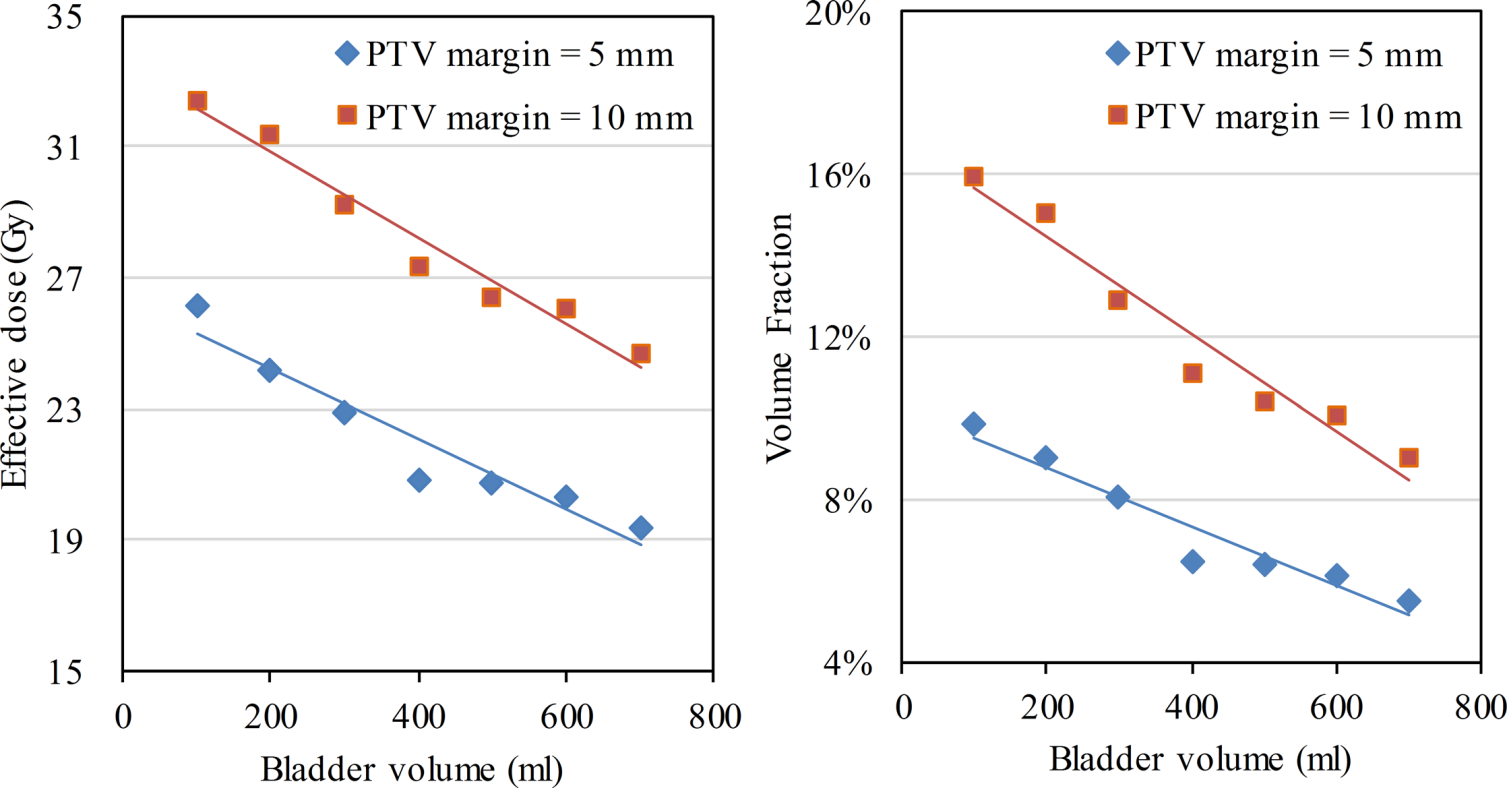
Effective dose and volume fraction received more than 50 Gy of the bladder in radiotherapy with different PTV margins.

*D*_eff_ of the bladder and V50 of the bladder wall (V50b) are shown in Fig 5. As the PTV margin increased, a larger volume of the bladder wall was irradiated: when the PTV margin was 5 mm, *D*_eff_ was 19.4–26.1 Gy and V50b was 5.5–9.8%, and when the PTV margin was 10 mm, *D*_eff_ was 24.7–32.4 Gy and V50b was 9.0–15.9%. When the bladder volumes were constant, an extension of the PTV margin from 5 mm to 10 mm increased *D*_eff_ and V50b by 5.3–7.2 Gy and 3.5–6.1%, respectively.

As the bladder volume increased, the thickness of the bladder wall as well as the bladder wall volume located in the target volume decreased. When the intravesical volume was increased from 100 ml to 700 ml, the variation of *D*_eff_ and V50b showed a linear relationship with the change in bladder volume. As the intravesical volume increased at an interval of 100 ml, *D*_eff_ decreased by 1.1- (R^2^ = 0.92) and 1.3-Gy (R^2^ = 0.96) and V50b decreased by 0.74% (R^2^ = 0.93) and 1.0% (R^2^ = 0.95) at 5 and 10 mm PTV margins, respectively.

**Fig 6:**
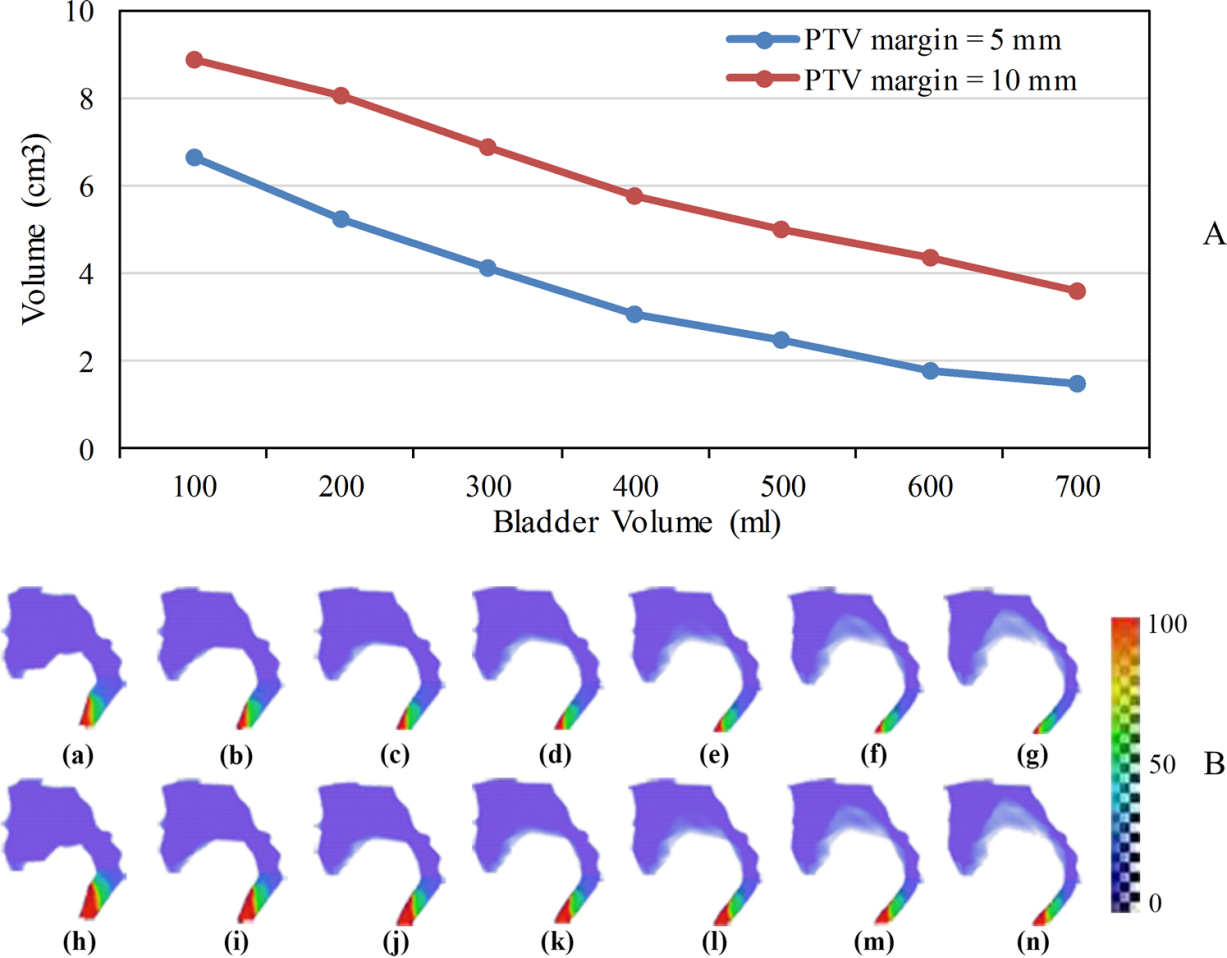
(A) Intestines volume that received more than 50 Gy for different PTV margins and (B) an intuitive representation of the distribution of the dose (Gy) to the intestines when intravesical volume varied from 100 ml to 700 ml with 5 (a–g) and 10 mm (h–n) PTV margins.

V50 of the intestines (V50i) was used to represent the dose to the small intestine and the rectum. V50i decreased by 0.9 cm^3^ per 100-ml increase in intravesical volume (R^2^ > 0.96) (Fig 6).

## Discussion

This study confirmed that increasing bladder volume can facilitate the displacement of OAR beyond the radiotherapy target volume and decrease the effective dose delivered to the bladder wall and the intestines. However, a large bladder volume causes an unstable prostate position, and clinical CT-based investigations have been conducted to predict the pattern of prostate movement [4,25,26]. The prostate motion in this study was more gradual than that in the report by Zellars *et al*. [4], who observed a 3-mm prostate displacement caused by a 100-ml change in bladder volume. The difference may be attributed to the constraint of the bottom of the pelvic model in the FE analysis, which prevented the prostate from moving down. The displacement of the bladder wall in each direction obtained with the FE analysis is consistent with the clinical findings of Pinkawa *et al*. [27]. Overall, the FE analysis provides a convenient, nonradioactive, and universal method of predicting organ deformation and displacement.

This study revealed the linear relationship between the dose to the bladder wall and the intravesical volume. If the PTV margin is constant, *D*_eff_ of the bladder wall increases by 1.1–1.3 Gy when the intravesical volume decreases by 100 ml. However, the PTV margin can be reduced to ≤6 mm by using the IGRT technique [28]. In the simulations with different PTV margins and intravesical volumes, the OAR dose was similar when the intravesical volumes were 100 and 400 ml with 5-mm and 10-mm PTV margins, respectively.

Our findings are comparable to those of Moiseenko *et al*. [1], who conducted a treatment planning study in 21 prostate cancer patients. The mean bladder volumes were 354.3 and 118.2 cm^3^ when the bladder was full and empty, respectively, and the PTV was generated by adding a 10-mm 3D margin to the prostate. The average *D*_eff_ of the bladder was similar to the dose, which was determined from the linear dose/volume relationship identified in the present study, when the bladder was full (29.0 Gy vs. 28.8 Gy) but was more serious than our results when the bladder was empty (49.3 Gy vs. 31.9 Gy). The difference may be caused by two factors. First, the VCH phantom in our study was a 24-year-old male cadaver without prostatic hypertrophy protrusion into the bladder [29], which is commonly observed in elderly patients and increases the volume of the bladder wall in the irradiation region when the bladder is empty. Second, *D*_eff_ reported by Moiseenko *et al*. [1] was not obtained from a single patient; thus, it could have been affected by individual differences in the position and shape of the pelvic organs.

The dosimetry of the intestines revealed that V50i increased by 0.8 cm^3^ when the intravesical volume decreased by 100 ml, and the V50 region of the intestines was mainly located in the rectum but rarely found in the small intestine. This finding is in line with the results of Moiseenko *et al*. [1], who reported that only 6 out of 21 patients received more than 50 Gy in the small intestine, covering an area of 2.5–30 cm^3^, and 8 patients did not receive more than 15 Gy in any part of the small intestine when the bladder was empty. However, a retrospective study indicated that treatments planned for patients with an empty bladder resulted in increased acute GI toxicity, which was most likely because of an increase in the radiation dose delivered to the small intestine [30]. We believe that the difference may be attributed to prostatic hypertrophy, which brings a larger portion of the bladder wall, along with a greater volume of the small intestine, into the target volume.

Computational phantoms have been employed for dose evaluation in CT, positron emission tomography, and other clinical procedures that involve radiation [31]. This study attempted to utilize a computational phantom in radiotherapy and generally, the dosimetry result predicted by this study is consistent with reported clinical results. Therefore, FE analysis of computational phantoms is capable of predicting organ motion and can be extented to model other deformable organs (such as the lungs, stomach, and heart) that can also profoundly affect the radiotherapy dosimetry. Nevertheless, phantoms representing elderly individuals as well as those with various pathologies have yet to be developed. Therefore, although computational anthropomorphous phantom is a promising tool in clinical radiation protection, this approach currently has obvious limitations. Efforts should be intensified to develop a technology that would allow rapid construction of accurate patient-specific computational phantoms.

## Conclusions

This study confirmed in a quantitative manner that intravesical volume is a dominant factor that affects OAR dose. With the development of tumor-tracking technology, OAR toxicity in prostate radiotherapy could be minimized with both a filled and an empty bladder. Bladder as well as urinary tract characteristics should be evaluated in each patient to determine the optimal preloading volume for prostate radiotherapy. Furthermore, FE analysis is a feasible method to predict organ deformation and displacement. Therefore, the effects of organ motion on radiation dose can be predicted provided that a proper computational anthropomorphous phantom is available.
